# Effect of Dexamethasone, Insulin and EGF on the Myogenic Potential on Human Endometrial Stem Cell

**Published:** 2014

**Authors:** Hora Jalali Tehrani, Kazem Parivar, Jafar Ai, Abdolmohammad Kajbafzadeh, Reza Rahbarghazi, Mehrdad Hashemi, Majid Sadeghizadeh

**Affiliations:** a *Depatment of Biology, Science and Research Branch Islamic Azad University Tehran Iran. *; b *Department of Tissue Engineering, Faculty of Advance Technology, University of Medical Sciences, Tehran, Iran. *; c *Paediatric Urology Research Center, Department of Urology, Children's Hospital Medical Center, Tehran University of Medical Sciences,Tehran, Iran. *; d *Umbilical Cord Stem Cell Research Center, Tabriz University of Medical Sciences, Tabriz, Iran. *; e *Department of Medical Genetics. Tehran Medical Sciences Branch .Islamic Azad University Tehran, Iran. *; f *Department of Genetics, Faculty of Biological Sciences, Tarbiat Modares University, Tehran, Iran.*

**Keywords:** Dexamethasone, Endometrial stem cell, Myogenic potential, Differentiation

## Abstract

Human endometrium contains mesenchymal stem cells (eMSC) which have the ability to differentiate into three cell lineages and the potential in therapeutic applications. We hypothesize that using environmental induction in culture media such as dexamethasone, human recombinant insulin and human epidermal growth factor (hEGF) can differentiate endometrial stem cells into myoblast. These agents have a broad range of effects in myoblast differentiation *in-vitro*. We used immunohystochemistry analysis and RT –PCR to evaluate the presence of skeletal muscle - specific proteins some of which are expressed in the early stage of differentiation including myoD and Desmin which expressed at later stages of differentiation. In conclusion eMSC can differentiate in culture media which contains above mentioned factors and use for therapeutic purpose in muscular degenerative disease.

## Introduction

Glucocorticoid treatment is highly effective for inflammatory myopathies and many types of muscular dystrophy such as Duchenne's muscular dystrophy (DMD). Therapeutic effects of glucocorticoids are often attributed to their potent anti-inflammatory activity studies in DMD patients indicated that glucocorticoid therapy reduced muscle inflammation and muscle proteolysis (1), while increasing myogenic repair and myoblast proliferation (2). Human endometrium is a dynamic tissue which consists of the functionalis and basalis layers undergo about 400 cycles of regeneration, proliferation, differentiation, tissue breakdown and shedding (3, 4).Human endometrium contains adult stem cells with multipotent activity(5) .These cells express sufrace markers similar to the phenotype of bone marrow and mesenchymal stem cell of adipose tissue including CD 90,CD105 but not CD31(endothelial) , CD34(hematopoetic stem cell and endothelial)(6). Most endometrial mesenchymal stem cell (eMSC) posses a remarkable myogenic capacity wich can be used for treatment of Duchenne muscular dystrophy or cardiac myocardial infarction models (7). Myogenesis is regulated by a family of transcription factors (myogenic regulatory factors (MRFs), including MyoD, Myf5, myogenin, and MRF4. MyoD, belongs to the basic helix-loop-helix protein superfamily and is expressed in proliferating, undifferentiated myoblasts, exhibiting a nuclear localization (8), whereas Desmin is required for terminal differentiation (9). Previous study shows that treatment with low doses of dexamethasone resulted increase in mRNA levels of the myogenic factor MyoD (2). In one study researchers were expanded human muscle precursor cells in culture media supplementing with insulin and dexamethasone at collagene coated dishes (10). 

 The major purpose of this work was to investigate the effect of signaling molecules such as dexamethasone, insulin, EGF in myogenic differentiation of eMSC and their use in medical applications.

## Experimental

*Isolation and culture of cells from human endometrium*

Endometrial biopsies were obtained from ovulating women without any endometrial disease. Informed written consent from describing the protocol and aims of the study was obtained from each patient. The biopsies were washed with HBSS (14025.Invitrogen .USA) and were finely minced and digested in HBSS and collagenase typeI (c0130.Sigma Aldrich.USA). The suspension was filtered through 70µm sieve (352340-BD Falcon) cells were then centrifuged for about 15-20 min. Mononuclear cells separated by using Ficoll – plaque gradient. Subsequently cells were cultured in low- glucose DMEM medium (31600-091. Invitrogen.USA) with10%FBS (10270.Invitrogen.USA), 1% pen/strep (15140.Invitrogen.USA, 1% amphotericin B (15290018.Invitrogen.USA) incubated at 37 ºC, 5% CO_2_ and allowed to attach for 24 h.

*Myogenic differentiation*

After endometrial stem cells were seeded at 5,000-15,000 cell/cm^2 ^in collagen coated dishes and DMEM/LG supplemented with %10 FBS supplementary agents: 0.5 mg/mL BSA (a2153.Sigma Aldrich), 0.1 µmol Dexamethasone (D1159.100MG. Sigma .USA), 10 µg/mL human recombinant insulin (12643-50MG.Sigma.USA), 10 ng/mL human epidermal growth factor (E9644.2MG.Sigma.USA) were added. Cells were also cultured concurrently for 2 weeks in 5% FBS and %5 horse serum (sh30074.03.Hyclon) containing medium without differentiation factor as control. Then cells were harvested for RNA extraction and immunocytochemistry using specific antibodies.

*Immunocytochemistry analysis*

After 16 day treatment cells were fixed with %4 paraformaldehyde for 20 min and permeablized with % 0.2 PBS –Triton X100(1.8903.100.Merk). Non specific binding was avoided by using %5 PBS-BSA for 1 h in 37 ºC. Cells were then incubated overnight at 4 ºC with these human specific antibodies: rabbit polyclonal anti-desmin (D8281.Sigma Aldrich), mouse monoclonal anti myoD (554130.BD pharmingen). After rinse in %5 PBS-Tween 20 for 5 min cells were incubated with FITC conjugated anti mouse IgG (ab 6785.abcam) or FITC conjugated anti-rabbit IgG (F9887.Sigma Aldrich) for 1 h at room temperature and again washed with PBS –tween 20(three times, 5 min each). Nuclei were stained with PI and image was taken by inverted fluorescent microscope (BX41.Olympus. Japan).

*Total RNA isolation and reverse transcription polymerase chain reaction*

Total RNA extracted with Qiazol Lysis Reagent kit (79306.Qiagen, Texas, USA) from differentiated and control cells. RNA quality was assessed by spectrophotometry and the purity was analyzed by the 260:280 absorbance ratios. First strand complementary DNA was synthesized using Bio RT cDNA first strand synthesis kit (BSB09M1.Bioe technology .Hangzhou.Japan) for reverse transcriptase-PCR complementary DNA reaction volume were 2 µL for each PCR assay. The primers used in this study were constructed by Oligo -7 software as follows: myoD F: CGCCATCCGCTATATCGAGG R: CTGTAG TCCATCATGC CGTCG, desmin F: GAGACCATCGCGGCTAAGAAC R: GTGTAGG ACTGGATCTGGTGT

HPRT F: CCTGGCGTCGTGATTAGTGAT R: AGACGTTCAGTCCTGTC CATAA

Complementary DNA was amplified using Real Q PCR 2×master mix kit (250527. Ampliqon) and primers with a total of 45 cycles PCR product were analyzed using %3 agarose gel electrophoresis and SYBER. 

## Results

*Morphological observation*

**Figure 1:**
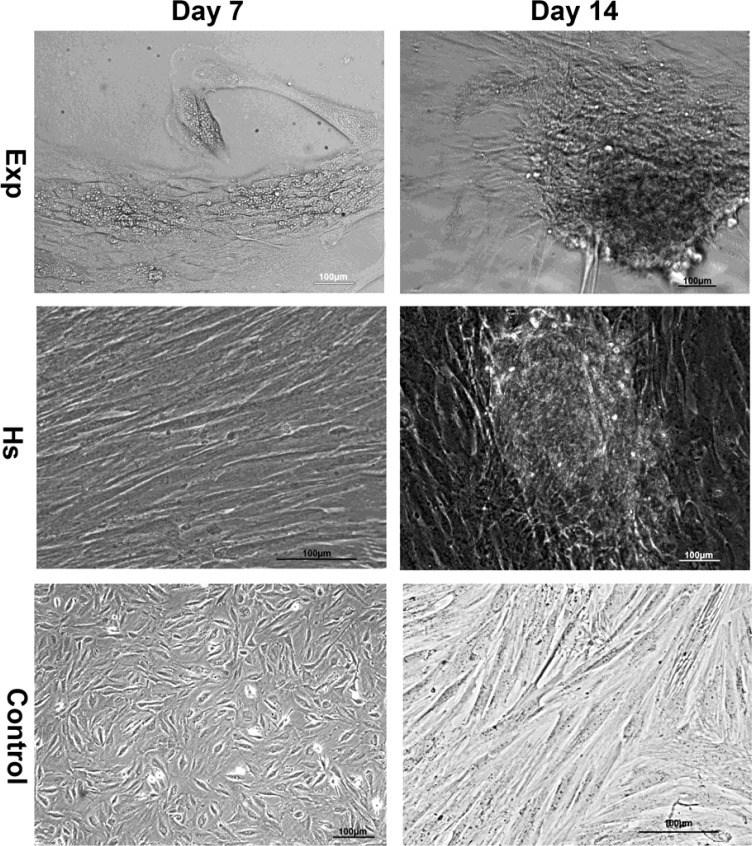
Photomicrograph of human endometrial stem cell in control group after 2-3 passage without any induction after 7 and 14 days (control).Cells were aggregated after 14 days when exposed to induction media (Exp) and horse serum (HS).

In order to study the specific effects of induction media composed dexamethasone on endometrial stem cells we cultured them under myogenic differentiation condition for about 16 days. We investigated the process of cell differentiation by Nikon ECLIPSE light microscopy (TS 100-F –Japan) and took photographs of cells with Nikon Digital Sight (Ds-L2) monitor. Stem cells were exposed to differentiation medium contained dexamethasone and in horse serum group. The proliferated quickly up to 80-90% and gradually began to aggregate but in control group cell proliferation and aggregation were normal according to the doubling time. These results indicated that cells which were influenced by differentiation medium and horse serum migrate in bottom of the culture plate and aggregated and they began to differentiate (Figure1).

*Immunocytochemical analysis*

**Figure 2:**
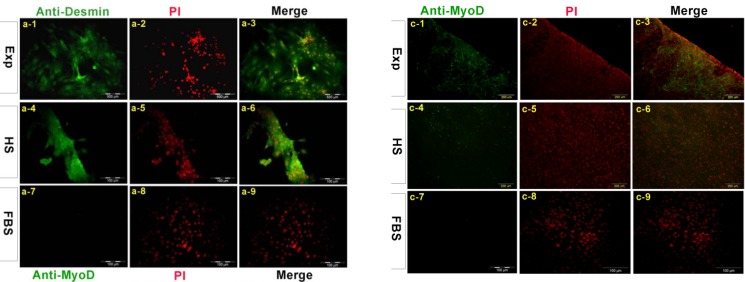
Immunolocolization of myogenic markers in EXP, HS and FBS group. Cells were stained with anti-desmin antibody (a1-a9). Desmin was detected in both nucleus and cytoplasm in treated cell with dexamethasone. B1-b9 indicates staining with antibody against Troponin I, in EXP and HS group troponin I was distributed in the whole cell. Cells were stained with myoD antibody weakly (c1-c9) MHC was expressed in cytoplasm and nucleus of cells in EXp group and expressed in HS group weakly (d1-d9). All nucleus were stained with PI

To evaluate the expression of muscle specific markers we used immunocytochemical analysis. Cells were harvested after 16 days. Differentiated cells in experimental group exposed to dexamethasone expressed Desmin protein in a large quantity in the cytoplasm of cells in the experimental group and less in horse serum group but not in control group. The average amount of MyoD protein was expressed in above mentioned groups (Figure 2).

*RT –PCR analysis*

**Figure 3:**
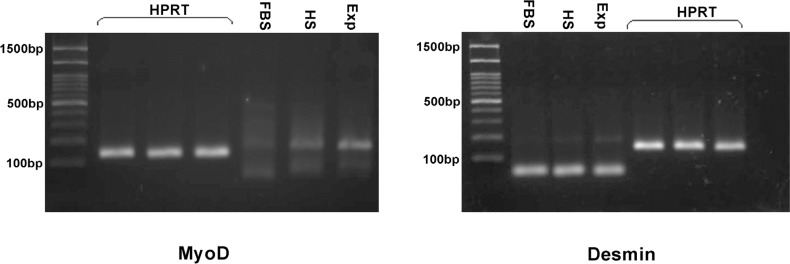
Gel of PCR reaction products of the myogenic markers myoD and desmin in control group (FBS), horse serum group (HS), induction media with dexamethasone (Exp).Expression of desmin and myoD in treated cells after 16 days apparently increased when compared with control group and it is weak in HS group. HPRT was used as the housekeeping gene control. PCR size marker is shown at the left of each panel. Arrows indicate 100 bp (lower) and 500 bp (upper) molecular weight marker bands

To investigate the effect of media composed dexamethasone on expression of myogenic markers, RT-PCR was performed. In experimental group, expression of MyoD and Desmin was detected specifically whereas in horse serum group the protein expression was reduced and in control group was not found implying that environmental factors that we added to the culture medium induced myogenic differentiation (Figure3). 

## Discussion

In our study we used several inducing substances such as insulin, growth factor, glucocorticoid hormones to demonstrate the myogenic potential of endometrial stem cells. Glucocorticoids have a broad range of effects in myoblast differentiation *in-vitro* (2). They have been shown to potentiate the mitogenic actions of insulin- like growth factor in multiple cultured cell types (11). They regulate cell survival, proliferation, and differentiation by modulating the expression of a variety of molecules and signaling cascades, in many cells and tissues (8). Dexamethasone treatment accelerated and increasesd the myotube fusion and terminal muscle differentiation program in myocyte (2). We expanded the endometrial epithelial and stromal cell in culture in order to identify the specific markers and demonstrated myogenic differentiation. According to previous studies these mesenchymal stem cell (MSC) strongly express pluripotent embryonic stem cell marker Oct4 (6, 12-14) and some mesenchymal stem cell markers such as CD90,CD 105, while lacking endothelial hematopoietic stem cell markers such as CD31,CD34 (6, 14-16). It is suggested that these multipotent MSC have differentiation potential into 3 cell lineage (6, 17). Previous studies have shown that insulin and insulin- like growth factors (IGF-I) stimulate proliferation and differentiation of skeletal muscle cells (18, 19). Accordingly dexamethasone treatment can afford expression of transcription factor MyoD and muscle structural protein MHC. It is well known that treatment with the artificial dexamethasone induces an increase in skeletalmuscle Na^+^,K^+^ pump content of 20–60%  (20). It also has a positive effect on protein synthesis and a protective effect on muscle cells (10). Glucocorticoids have beneficial on strength in muscular dystrophy disease which associated with an increase in muscle mass (21). According to these findings and with regard to the effect of insulin on chondrogenic , osteogenic and adipogenic differentiation (22), also epidermal growth factor which stimulate cell division and has mitogenic effects on muscle cell in culture(10, 23), we used also dexamethasone in culture media to investigate the proceeding of skeletal muscle differentiation *in-vitro* (10, 23). Skeletal myogenesis is a developmental cascade that involves the regulatory MyoD gene family that determines the progress of multipotential mesodermal stem cell into myogenic lineage. The MyoD family is one of the basic helix- loop- helix transcription factors that directly regulate myocyte cell specification, differentiation and express at the early stage of myogenic differentiation (9, 24, 25). Desmin is an intermediate filament found near the Z line in sarcomeres (18). In conclusion eMSC can differentiate into skeletal muscle cell when they expose to specific signaling molecules and have potential for use in medical applications.

## References

[1] Zhang J, Gao W, Yan S, Zhao Y (2012). Effects of space flight on the chemical constituents and anti-inflammatory activity of licorice (Glycyrrhiza uralensis Fisch). Iran. J. Pharm. Res.

[2] Belanto JJ, Diaz-Perez SV, Magyar CE, Maxwell MM, Yilmaz Y, Topp K, Boso G, Jamieson CH, Cacalano NA, Jamieson CA (2010). Dexamethasone induces dysferlin in myoblasts and enhances their myogenic differentiation. Neuromuscul. Disord.

[3] Zemelko VI, Grinchuk TM, Domnina AP, Artzibasheva IV, Zenin VV, Kirsanov AA, Bichevaia NK, Korsak VS, Nikolsky NN (2012). Multipotent mesenchymal stem cells of desquamated endometrium: Isolation, characterization, and application as a feeder layer for maintenance of human embryonic stem cells. SP MAIK Nauka/Interperiodica.

[4] Maruyama T, Masuda H, Ono M, Kajitani T, Yoshimura Y (2010). Human uterine stem/progenitor cells: their possible role in uterine physiology and pathology. Reproduct..

[5] Gargett CE, Schwab KE, Zillwood RM, Nguyen HP, Wu D (2009). Isolation and culture of epithelial progenitors and mesenchymal stem cells from human endometrium. Biol Reprod.

[6] Gargett CE, Masuda H (2010). Adult stem cells in the endometrium. Molecul. Human Reproduct.

[7] Ding D-C, Shyu W-C, Lin S-Z (2011). Mesenchymal Stem Cells. Cell Transplantat.

[8] Bruscoli S, Donato V, Velardi E, Di Sante M, Migliorati G, Donato R, Riccardi Carlo (2010). Glucocorticoid-induced leucine zipper (GILZ) and long GILZ inhibit myogenic differentiation and mediate anti-myogenic effects of glucocorticoids. Journal of Biological Chemistry.

[9] De Bari C, Dell'Accio F, Vandenabeele F, Vermeesch JR, Raymackers JM, Luyten FP (2003). Skeletal muscle repair by adult human mesenchymal stem cells from synovial membrane. J. Cell Biol.

[10] Eberli D, Soker S, Atala A, Yoo JJ (2009). Optimization of human skeletal muscle precursor cell culture and myofiber formation in-vitro. Methods.

[11] Giorgino F, Smith RJ (1995). Dexamethasone enhances insulin-like growth factor-I effects on skeletal muscle cell proliferation Role of specific intracellular signaling pathways.. J. Clin. Invest.

[12] Gargett CE, Chan RW, Schwab KE (2008). Hormone and growth factor signaling in endometrial renewal: role of stem/progenitor cells. Mol. Cell Endocrinol.

[13] Rodrigues MC, Glover LE, Weinbren N, Rizzi JA, Ishikawa H, Shinozuka K, Tajiri N, Kaneko Y, Sanberg PR, Allickson JG, Kuzmin-Nichols N, Garbuzova-Davis S, Voltarelli JC, Cruz E, Borlongan CV (2011). Toward personalized cell therapies: autologous menstrual blood cells for stroke. J. Biomed. Biotechnol.

[14] Meng X, Ichim TE, Zhong J, Rogers A, Yin Z, Jackson J, Wang H GeW, Bogin V (2007). Chan KW and Thebaud B Riordan, N. H. Endometrial regenerative cells: a novel stem cell population. J. Transl. Med.

[15] Cervelló I, Gil-Sanchis C, Mas A, Delgado-Rosas F, Martínez-Conejero JA, Galán A, Martínez-Romero A, Martínez S, Navarro I, Ferro J, Horcajadas JA, Esteban FJ, O'Connor JE, Pellicer A, Simón C (2010). Human Endometrial Side Population Cells Exhibit Genotypic, Phenotypic and Functional Features of Somatic Stem Cells. PLoS ONE.

[16] Chan RWS, Schwab KE, Gargett CE (2004). Clonogenicity of human endometrial epithelial and stromal cells. Biol. Reproduct.

[17] Schwab KE, Gargett CE (2007). Co-expression of two perivascular cell markers isolates mesenchymal stem-like cells from human endometrium. Human Reproduct.

[18] Conejo R, Valverde AM, Benito M, Lorenzo M (2001). Insulin produces myogenesis in C2C12 myoblasts by induction of NF-kappaB and downregulation of AP-1 activities. J. Cell Physiol.

[19] Tabeefar H, Beigmohammadi MT, Javadi MR, Abdollahi M, Mahmoodpoor A, Ahmadi A, Honarmand H, Najafi A, Mojtahedzadeh M (2011). Effects of Pantoprazole on Systemic and Gastric Pro-and Anti-inflammatory Cytokines in Critically Ill Patients. Iran. J. pharm. Res.

[20] Nordsborg N, Goodmann C, McKenna MJ, Bangsbo J (2005). Dexamethasone up‐regulates skeletal muscle maximal Na+, K+ pump activity by muscle group specific mechanisms in humans. J. physiol.

[21] Rifai Z, Welle S, Moxley R, Lorenson M, Griggs RC (1995). Effect of prednisone on protein metabolism in Duchenne dystrophy. American J. Physiol. Endocrinol. Metabol.

[22] Malafaya PB, Oliveira JT, Reis RL (2009). The Effect of Insulin-Loaded Chitosan Particle–Aggregated Scaffolds in Chondrogenic Differentiation. Tissue Engineering Part A.

[23] McFarland DC (1992). Cell culture as a tool for the study of poultry skeletal muscle development. J. Nut.

[24] Gang EJ, Jeong JA, Hong SH, Hwang SH, Kim SW, Yang IH, Ahn C, Han H, Kim H (2004). Skeletal myogenic differentiation of mesenchymal stem cells isolated from human umbilical cord blood. Stem. Cells.

[25] Cui C-H, Uyama T, Miyado K, Terai M, Kyo S, Kiyono T, Umezawa A (2007). Menstrual Blood-derived Cells Confer Human Dystrophin Expression in the Murine Model of Duchenne Muscular Dystrophy via Cell Fusion and Myogenic Transdifferentiation. Molecular Biol. Cell.

